# Image-guided Percutaneous Radiofrequency Ablation for Osteoid Osteoma: Experience from a Developing Nation

**DOI:** 10.7759/cureus.5633

**Published:** 2019-09-12

**Authors:** Mustafa Belal Hafeez Chaudhry, Basit Salam, Kumail Khandwala, Raza Sayani, Azeemuddin Muhammad, Tanveer U Haq

**Affiliations:** 1 Radiology, Aga Khan University Hospital, Karachi, PAK

**Keywords:** osteoid osteoma, radiofrequency ablation, computed tomography, image-guided

## Abstract

Objective

The purpose of this study is to report our experience in using image-guided percutaneous radiofrequency ablation (RFA) for the treatment of osteoid osteoma (OO) and the subsequent duration of pain relief over a period of about six years (May 2013-March 2019; 70 months) at a tertiary-care hospital in a developing nation.

Methods

A retrospective study was performed at the radiology department of Aga Khan University, Karachi, Pakistan. All patients who had undergone image-guided percutaneous RFA for OO between May 2013-March 2019 were included. All cases had been performed with CT-guidance under general anesthesia, with an additional local anesthesia injection also administered to the patients. A soloist needle had been used for RFA. The primary success rates, complications, symptom-free intervals, and follow-ups were evaluated.

Results

In total, 15 patients (11 males, 4 females) of a mean age of 13.93 years (range: 5-25 years; median age: 14.5 years) with OO underwent image-guided percutaneous RFA during a period of 70 months. Eleven lesions were located in the femur, three in the tibia, and one in the humerus. The mean nidus size was 8.1 x 5.73 mm [range: (4.9-11.5) x (3.8-9.1) mm]. All patients were successfully treated and experienced resolution of pain in 2.36 months (range: 1-4 months). During the follow-up period (range: 3-40 months; mean: 13.85 months; median: nine months), none of the patients experienced any relapse or persistent symptoms. No major complications were reported.

Conclusion

Image-guided percutaneous RFA is a minimally invasive and safe treatment option with high efficiency and a high rate of technical success for the treatment of OO. The risk of recurrence is remote with all patients achieving independent recovery.

## Introduction

Osteoid osteoma (OO) is a relatively common, benign osteoblastic lesion that generally affects children and adolescents [1]. It accounts for 10% of all benign bone tumors and has a male-to-female predisposition ratio of 3:1 [2]. The most common and important clinical presentation is pain during the night that is dramatically relieved by salicylates or other nonsteroidal anti-inflammatory drugs (NSAIDs) [1, 3]. Other, less common, symptoms include growth disturbances, bone deformities, painful scoliosis, joint swelling, and contractures. A physical examination usually reveals localized tenderness without any signs of inflammatory disease. It is postulated that the pain may be mediated by the release of prostaglandins, which results in local vasodilatation [4, 5].

On plain films, OO is characterized by a circular or ovoid lucent nidus (usually less than 1.5 cm in diameter) with a variable degree of surrounding reactive sclerotic cortical thickening [3]. If sclerosis is extensive, it may interfere with the visualization of the radiolucent nidus on plain radiographs. CT is the most sensitive and specific option in the diagnosis and localization of OO, and thus the imaging modality of choice [2].

Different treatment strategies for OO have been implemented, including conservative medical treatment, surgery, and percutaneous intervention. Conservative medical therapy is now considered undesirable as it necessitates a prolonged period of therapy and long-term use of anti-inflammatory drugs. Surgery is considered curative; however, some intramedullary and subperiosteal lesions are difficult to localize intraoperatively as they may not demonstrate significant sclerosis and the cortex overlying the site may appear normal during the procedure. Surgery is also challenging for lesions smaller than 1 cm in diameter as it may lead to significant unnecessary bone resection [6]. Therefore, CT-guided percutaneous RFA is an effective technique that has been universally acknowledged as a minimally invasive, safe, and cost-effective cure for OO. The purpose of this study is to describe our experience in using RFA for the treatment of OO, its success rate, the duration of the pain relief it provided, and potential complications encountered over a period of about six years (May 2013-March 2019; 70 months) at a tertiary-care hospital in a developing nation.

## Materials and methods

A retrospective study was performed at the radiology department of Aga Khan University, Karachi, Pakistan, after gaining approval from the institutional ethical review committee (ERC). All patients who underwent image-guided percutaneous RFA for OO between May 2013 to March 2019 were included.

**Figure 1:**
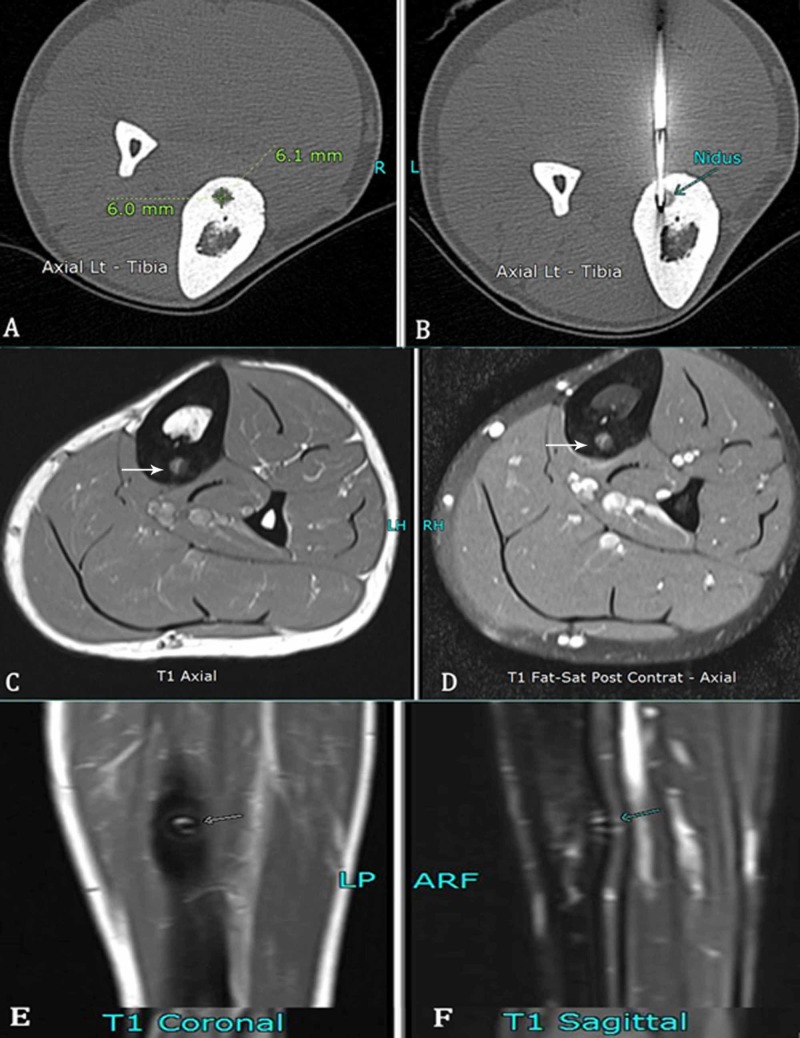
Various imaging findings of a 24-year-old male with an osteoid osteoma in the left tibia. A) Unenhanced CT axial section showing osteoid osteoma in the tibia measuring 6.1 x 6 mm. B) Soloist needle in the nidus (arrow). C-F) Follow-up MRI after six months shows non-enhancing nidus (arrows), representing successful ablation.

**Figure 2:**
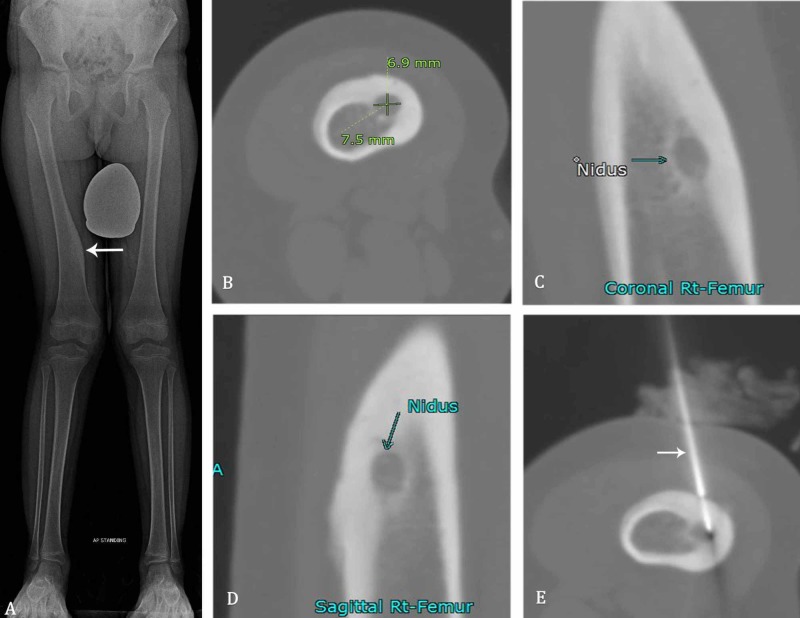
Various imaging findings of a six-year-old boy with an osteoid osteoma along the medial margin of the right femoral diaphysis. A) Scanogram radiograph showing osteoid osteoma in the medial right femur with associated cortical thickening (arrow). B-D) Nidus (arrows) is measured and well-demarcated on unenhanced CT-extremity images with surrounding cortical thickening. E) Soloist needle (arrow) is within the nidus and used for radiofrequency ablation.

The clinical diagnosis of OO was based on reports of severe bone pain, pain during the night, relief of pain after administration of NSAIDs, and on the basis of radiological features. Patients who were offered image-guided percutaneous RFA for OO were also required to meet specific imaging criteria, including the documentation of radiolucent nidus on plain film and CT imaging, MRI, or any of the two (Figures 1, 2). In addition, bony sclerosis, cortical thickening, and any periosteal reaction were also assessed. Patients who had undergone prior surgery or intervention were excluded. Patients or parents/guardians (in cases involving minors) were also informed regarding alternative treatment options. Informed consent was obtained from each patient or their parents before the procedure. Pre-procedural management included the assessment of complete blood cell count (CBC), blood clotting analyses (required platelet count: >75,000/mL; international normalized ratio: <1.5 mL), and physical fitness test to undergo general anesthesia. Patients were also examined for any evidence of local infection at the site of OO.

All cases were performed with CT-guidance under general anesthesia, with an additional local anesthesia injection also administered to the patients. All cases were performed by a board-certified interventional radiologist with at least five years of experience who was assisted by an interventional radiology fellow. Contiguous CT (Aquilion 640, Toshiba Medical Systems, Tokyo, Japan) scans with a section thickness of 1-5 mm were obtained to localize OO. Using the images, we adjusted the position of the patient’s limb and marked the skin at the planned access point. The skin was prepared and draped in the usual fashion. The lesion was accessed with a 14-gauge bone-biopsy needle (Bonopty by AprioMed, Uppsala, Sweden). Using this access made by the drill hole, the RF electrode (Soloist, Boston Scientific Corporation, Marlborough, Massachusetts, US) was introduced into the nidus (Figures 1B, 2E). Lesion ablation was subsequently performed using a standard protocol, which lasted between four-eight minutes.

Each patient was assessed and examined before discharge for bleeding, swelling, skin burn, neurovascular complications, and other procedure-related problems to assess the technical success rate.

## Results

**Figure 3:**
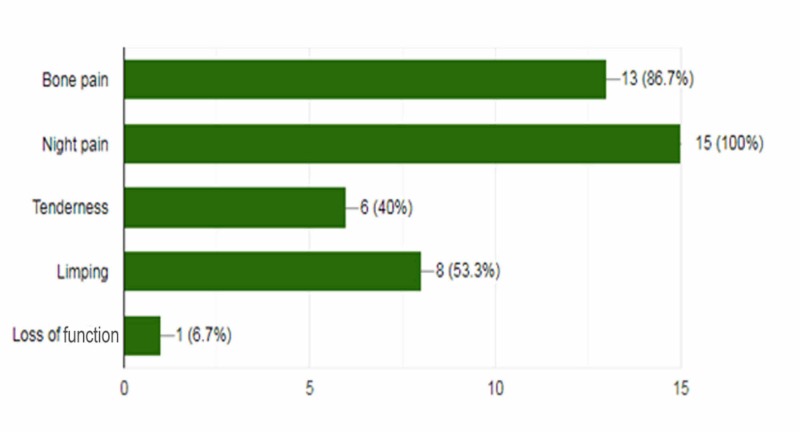
Frequency of presenting complaints in patients with osteoid osteoma.

In total, 15 patients (11 males, 4 females) underwent image-guided percutaneous RFA for OO during the period of 70 months (May 2013-March 2019). The mean age was 13.93 years (range: 5-25 years; median: 14.5 years). Pain during the night was the chief complaint from all patients, followed by bone pain and tenderness (Figure 3).

**Figure 4:**
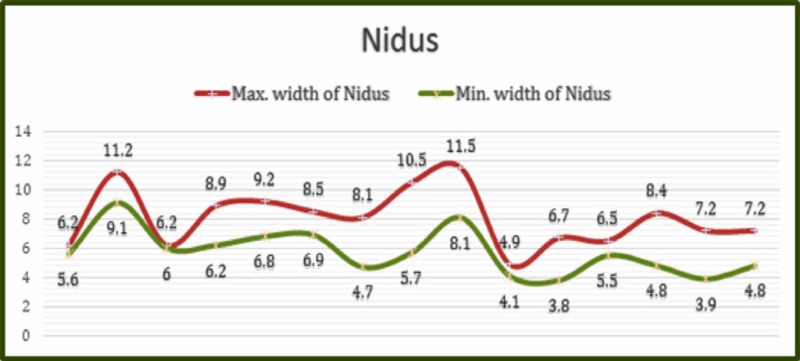
Relationship between the maximum and the minimum sizes (in mm) of the osteoid osteoma nidus.

A single lesion in a long bone was diagnosed and eventually treated in every patient. Out of 15 lesions, 11 were located in the femur, three in the tibia, and one in the humerus. The mean size of the OO nidus was 8.1 x 5.73 mm [(4.9-11.5) x (3.8-9.1) mm], with minimal variation between the maximum and the minimum dimensions of the nidus (Figure 4). The RFA procedure was classified as radiologically successful when the RFA electrode was successfully placed in the nidus, when there was pain relief, when there was no increase in the symptoms, or in the absence of recurrence. The procedure was technically successful in all of the cases.

**Figure 5:**
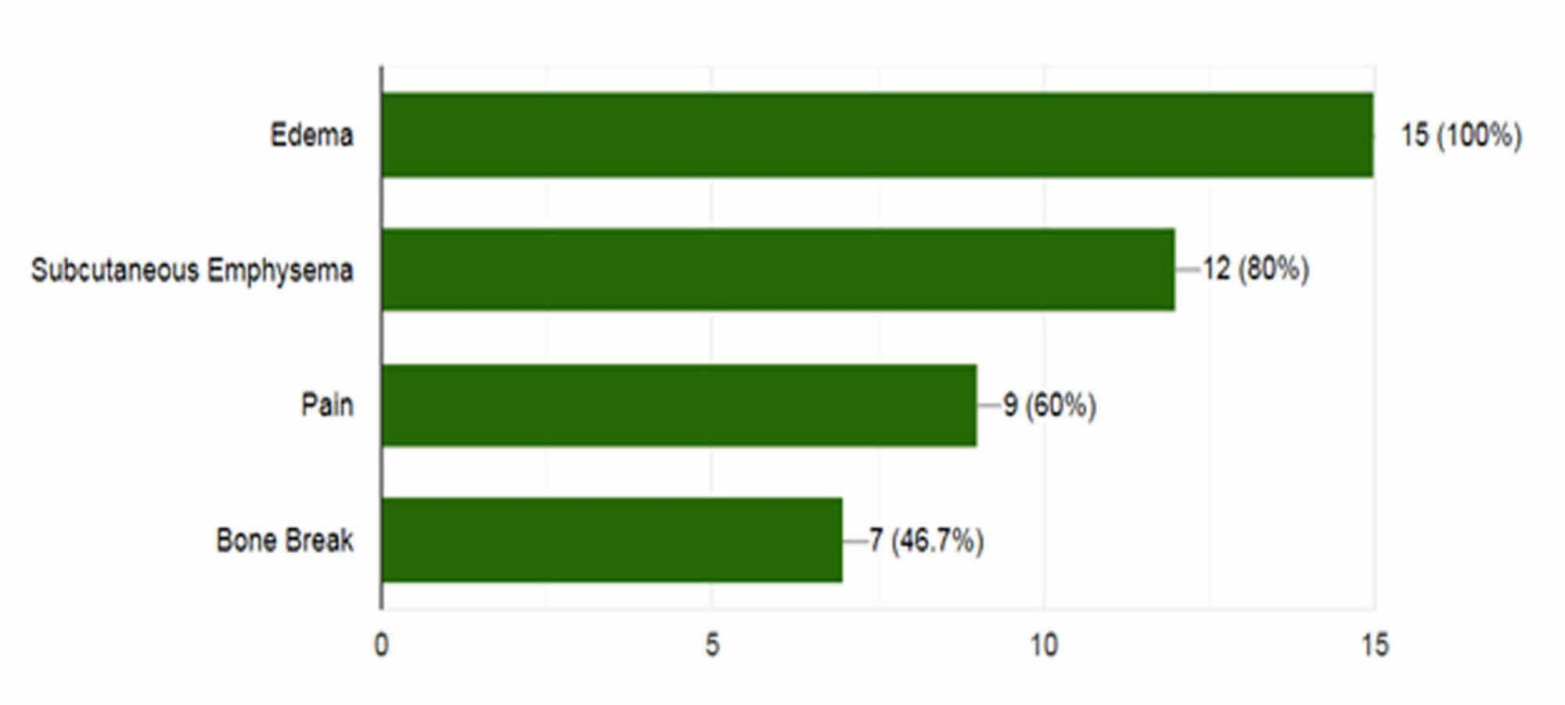
Frequency of peri-procedural local changes and minor complications.

The majority of the patients achieved complete pain relief within 10 days (1.5 weeks) after RFA (mean: 1.5 weeks; range: 0.5-3.5 weeks; median and mode: 1.5 weeks). All patients were capable of full weight-bearing after their procedure without any support. There was no major restriction in physical activity for any of the patients after the procedure. No major complications occurred during or after the procedure in any of the patient. However, a few minor peri-procedural complications were reported (Figure 5). All patients demonstrated subcutaneous edema after the procedure. A minimal cortical break was observed in seven (46.7%) cases, secondary to needle manipulation. No major fracture was reported, and there were no other procedure-related complications.

**Figure 6:**
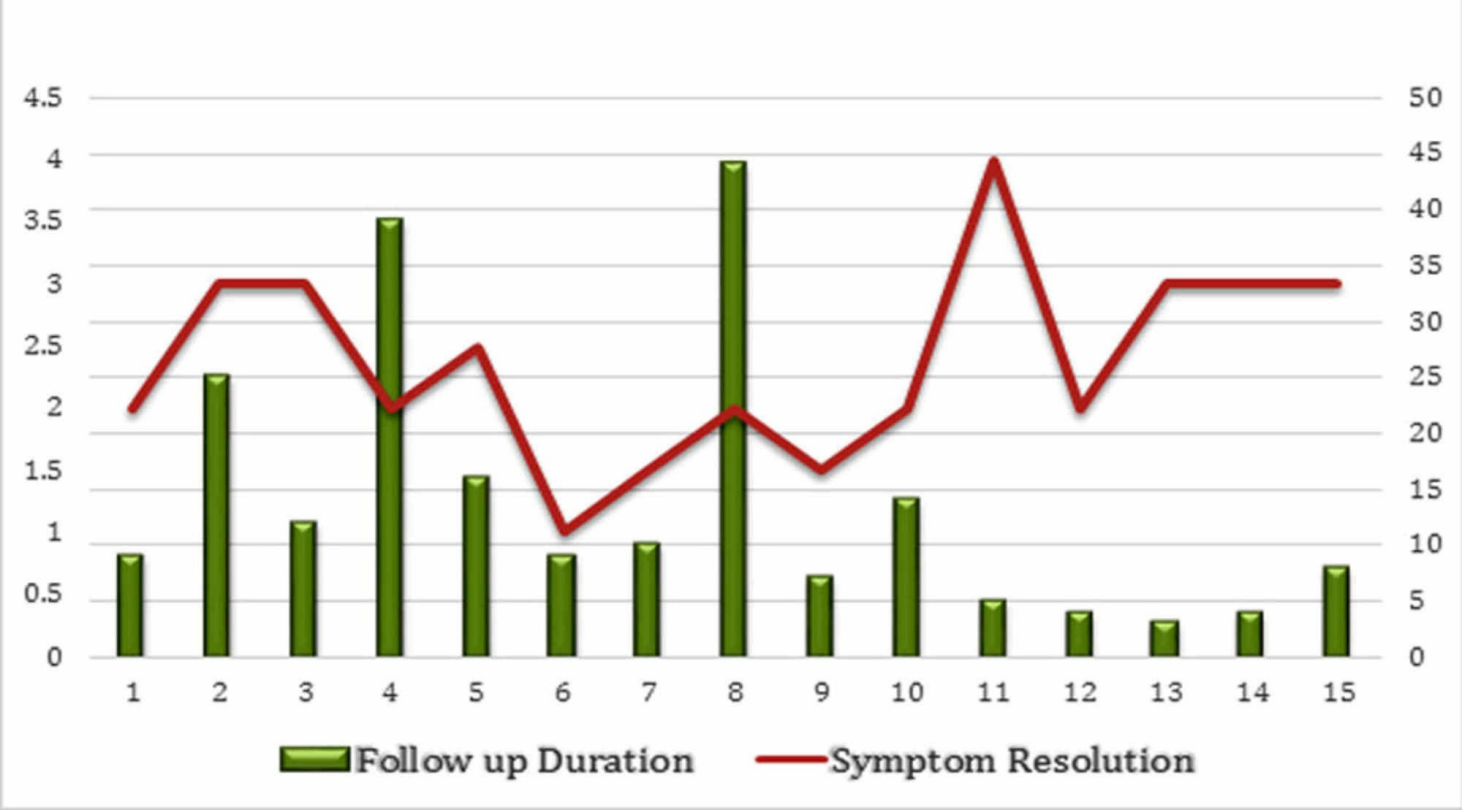
Relationship between the duration of symptom resolution and the follow-up period.

The mean follow-up duration was 13.85 months (range: 3-40 months; median: nine months). The graphical relationship between the duration of symptom resolution and the follow-up period is detailed individually below (Figure 6).

None of the patients experienced a persistence of symptoms or relapse during the follow-up period, and no recurrence was reported. Preliminary results of this study were presented in the 33rd Annual Conference of the Radiological Society of Pakistan in Karachi (Abstract: Mustafa Belal Hafeez Chaudhry, Basit Salam, Muhammad Azeemuddin, Raza Sayani, Tanveer ul Haq. Computed Tomography Guided Percutaneous Radiofrequency Ablation in Treating Osteoid Osteoma. Karachi, October 27-29, 2017). All the results are summarized below (Table 1).

**Table 1:** Summarized results with different study variables.

Study Group Demographics	
Variables	n (%)
Cases	15
Mean age	13.93 years (range: 5-25 years)
Males/females	7 (73%)/4 (27%)
Bone involved: femur/tibia/humerus	11 (73%)/3 (20%)/1 (7%)
Side involved: right/left	8 (53%)/7 (47%)
Duration of procedure	39.5 minutes (range: 25-50 minutes)
Symptom resolution (follow-up)	1.55 weeks (range: 0.5-3.5 weeks.)
Follow-up time (mean)	13.86 months (range: 3-44 months; median: nine months; mode: nine months)

## Discussion

Percutaneous RFA for OO was first reported by Rosenthal et al. in 1992 [6], demonstrating that image-guided percutaneous RFA is a safe and effective treatment for OO. In the present study, clinically successful treatment was performed in 100% of the cases, which is comparable to the success rates reported by many researchers, which range from 75% to 100%. It also compares favorably with other techniques such as surgical resection and laser ablation [7-9].

It is debatable whether or not a cooling RFA probe system should be used. While some researchers advocate its use to prevent osteonecrosis, others discourage it because of the increased risk of post-procedural pain and complications such as wound infections and burns. However, authentic and validated data in this regard is lacking [10-12]. We used a non-cooled system in all our patients as that is the one commercially available in Pakistan. We did not encounter any case of osteonecrosis in our patients who had follow-ups.

In cases of OO measuring over 1 cm in diameter, the use of two or more electrode positions is often necessary [3]. Previous reports have also suggested that the odds of technically unsuccessful percutaneous ablation increase if the nidus size is over 1 cm. We had three patients with a nidus size marginally greater than 1 cm (maximum: 1.15 cm). However, we were able to achieve technical success in all the three patients with the use of a single electrode, and without any residual or recurrent disease on follow-ups.

The major post-procedural complications reported in the literature include skin burns, skin and fat necrosis, soft-tissue infections, vasomotor instability, tendinitis, and hematoma [7-9]. However, we did not encounter any of these major complications in our patients during or after the procedure. These results are comparable to those of several previous reports of the successful use of image-guided percutaneous RFA for the treatment of OO [7-9]. Periprocedural soft- tissue edema was noted in all patients, which was relieved within three weeks. This has been reported by Murat Cakar et al. and is thought to be a result of prostaglandin discharge from OO during ablation [7]. All the cases of image-guided percutaneous RFA were performed under general anesthesia, as it assists to achieve a stable condition and helps in adequate pain control during the procedure. In addition, we also used local anesthesia in all patients for better periprocedural pain management. We also did not experience any anesthesia-related complications. Minor cortical breaks were observed during the RFA procedure in 46.7% of the patients, which did not require any additional treatment. In contrast to image-guided percutaneous RFA, percutaneous lesion resection leaves a bone defect that may be vulnerable to fracture and, in some cases, may necessitate internal fixation and bone grafting, and may also increase morbidity.

Caution is necessary when performing image-guided percutaneous RFA for spinal and hand OO to avoid nerve injuries. Relative contraindication for RFA in such cases is the presence of OO near a neurological structure within a distance of less than 5 mm, increasing the risk of spinal-cord damage by hyperthermia-induced cytotoxicity [13]. Additionally, RFA may also cause chondral damage due to thermal necrosis in juxta-articular lesions [14]. In our series, all lesions were located in the long bones of the extremities, which helped to avoid neural structures and major complications. We acquired immediate post-procedural CT sections of the target area to evaluate for any immediate complication. However, it is not considered mandatory to routinely perform CT after the procedure [3].

This study has a few limitations. One of the major limitations is the small sample size. It is followed by the absence of the histological confirmation of OO in any of the cases we studied. However, all of the patients did experience typical clinical symptoms of OO, and the diagnosis was confirmed by typical imaging findings in each case. Hence, the histological verification was unnecessary for the diagnosis of OO in all of the cases. Thirdly, there were no cases of spinal or small bone OO, denying us the opportunity to demonstrate the technical success rate of RFA for such lesions. Lastly, none of the lesions were large enough to utilize the dual-electrode technique.

## Conclusions

Image-guided RFA is a minimally invasive and safe option with high efficiency and a high rate of technical success for the treatment of OO and should be considered the treatment of choice in the current era. Our results also show that the risk of recurrence is remote with most patients achieving independent recovery.
